# Structured Pathway across the Transition State for Peptide Folding Revealed by Molecular Dynamics Simulations

**DOI:** 10.1371/journal.pcbi.1002137

**Published:** 2011-09-08

**Authors:** Lipi Thukral, Isabella Daidone, Jeremy C. Smith

**Affiliations:** 1 Interdisciplinary Center for Scientific Computing, University of Heidelberg, Heidelberg, Germany; 2 Department of Chemistry, Chemical Engineering and Materials, University of L'Aquila, Coppito, Italy; 3 UT/ORNL Center for Molecular Biophysics, Oak Ridge National Laboratory, Oak Ridge, Tennessee; University of North Carolina at Chapel Hill, United States of America

## Abstract

Small globular proteins and peptides commonly exhibit two-state folding kinetics in which the rate limiting step of folding is the surmounting of a single free energy barrier at the transition state (TS) separating the folded and the unfolded states. An intriguing question is whether the polypeptide chain reaches, and leaves, the TS by completely random fluctuations, or whether there is a directed, stepwise process. Here, the folding TS of a 15-residue β-hairpin peptide, Peptide 1, is characterized using independent 2.5 μs-long unbiased atomistic molecular dynamics (MD) simulations (a total of 15 μs). The trajectories were started from fully unfolded structures. Multiple (spontaneous) folding events to the NMR-derived conformation are observed, allowing both structural and dynamical characterization of the folding TS. A common loop-like topology is observed in all the TS structures with native end-to-end and turn contacts, while the central segments of the strands are not in contact. Non-native sidechain contacts are present in the TS between the only tryptophan (W11) and the turn region (P7-G9). Prior to the TS the turn is found to be already locked by the W11 sidechain, while the ends are apart. Once the ends have also come into contact, the TS is reached. Finally, along the reactive folding paths the cooperative loss of the W11 non-native contacts and the formation of the central inter-strand native contacts lead to the peptide rapidly proceeding from the TS to the native state. The present results indicate a directed stepwise process to folding the peptide.

## Author Summary

The folding dynamics of many small protein/peptides investigated recently are in terms of simple two-state model in which only two populations exist (folded and unfolded), separated by a single free energy barrier with only one kinetically important transition state (TS). However, dynamical characterization of the folding TS is challenging. We have used independent unbiased atomistic molecular dynamics simulations with clear folding-unfolding transitions to characterize structural and dynamical features of transition state ensemble of Peptide 1. A common loop-like topology is observed in all TS structures extracted from multiple simulations. The trajectories were used to examine the mechanism by which the TS is reached and subsequent events in folding pathways. The folding TS is reached and crossed in a directed stagewise process rather than through random fluctuations. Specific structures are formed before, during, and after the transition state, indicating a clear structured folding pathway.

## Introduction

In recent years, extensive investigation has been undertaken of the folding of small proteins and peptides that can be approximated as two-state folders. In these systems, only two stable populations are detected (folded and unfolded), separated by a single effective free energy barrier with only one kinetically important transition state (TS), the reaching of which can be considered as the rate limiting step [1]. The determination of the transition state ensemble (TSE) of these two-state systems and how it is traversed are, therefore, fundamental to our understanding of the physicochemical basis of protein folding [2].

Recent research on the TSE has been based on results from simulations [3]–[7], experimental mapping of site-specific contacts in the TSE by protein engineering [8], [9] and mixed experimental/computational approaches [10]–[20]. To obtain insight at residue-level detail, 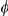-values are commonly used to investigate the formation of side-chain interactions in the TSE by mutating residues and assessing the effect on folding kinetics. While 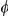-values do not, by themselves, directly provide structural information on the TS, they have been broadly used as structural restraints on a range of computational models (e.g., Go models [10], [20], Monte Carlo simulations [11], high-temperature, biased molecular dynamics (MD) [14], with both implicit solvent [15], [16] and all-atom MD representations [6]). However, it has been shown that not all conformations obtained in MD simulations by using the 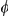-value as a restraint belong to the TS [21].

An alternative approach is to obtain the transition state ensemble from the maximum of the free energy surface projected onto selected folding coordinates, as has been done in many previous studies e.g., [6], [22]–[27]. The choice of proper reaction coordinates for protein folding is non-trivial, and whether a transition state identified using any given pair of progress variables corresponds to the transition state using another pair is not known a priori. In many of the above studies it was concluded that global coordinates based on the native topology of two-state proteins or peptides fully satisfy the criteria needed to accurately identify and describe the TSE. However, the need for further analysis of the folding/unfolding probabilities to validate the transition state ensemble has been emphasized [6], [14], [24], [28]. To address this, the identification of a TSE from free energy surfaces combined with validation of the TS structures found provides a secure way to characterize the transition state for folding.

Various hypotheses have been made concerning TS structures, varying from fully native [29] and partly native [17], [30] to denatured topologies [31]. Apart from the topology, there is debate as to whether the TS represents an ensemble with a single, unique nucleus [31]–[33] or a heterogeneous population of conformations, e.g., some with structure formed and others with structure absent [16], [20], [34]. On the one hand, it has been suggested that CI2 [32] and other proteins [35] contain specific contacts in the TS which are crucial to folding. On the other, heterogeneous TS theory involves the existence of multiple transition state ensembles through which parallel folding paths pass [20].

Arguably even more challenging than the structural characterization of the folding TS is the characterization of the TS from a dynamical point of view. Information on the mechanisms by which the TS can be reached and left along reactive folding trajectories, commonly named transition-path trajectories, is scarce. For this purpose, transition path sampling [4], [5] is often used, in which, given an initial reactive path, a shooting algorithm is employed to collect transition paths by perturbing the initial path. Although this method can generate an ensemble of transition paths, an initial reactive trajectory is needed, which is commonly generated through high-temperature unfolding simulations [5]. However, as the potential energy surface sampled at higher temperature is formally different from that visited at room temperature, unfolding through high temperature may well occur through pathways that are very different from the folding routes at lower temperatures. For example, a comparison between unfolding simulations performed at elevated temperature and folding simulations at room temperature has revealed that unfolding pathways lack important intermediates and often resemble œfast-track of folding [36]. Thus, an increase in temperature may actually change the folding process rather than simply accelerating it [37]. Hence, there remain significant advantages to characterizing folding processes using long-timescale simulations at room temperature.

**Figure 1:**
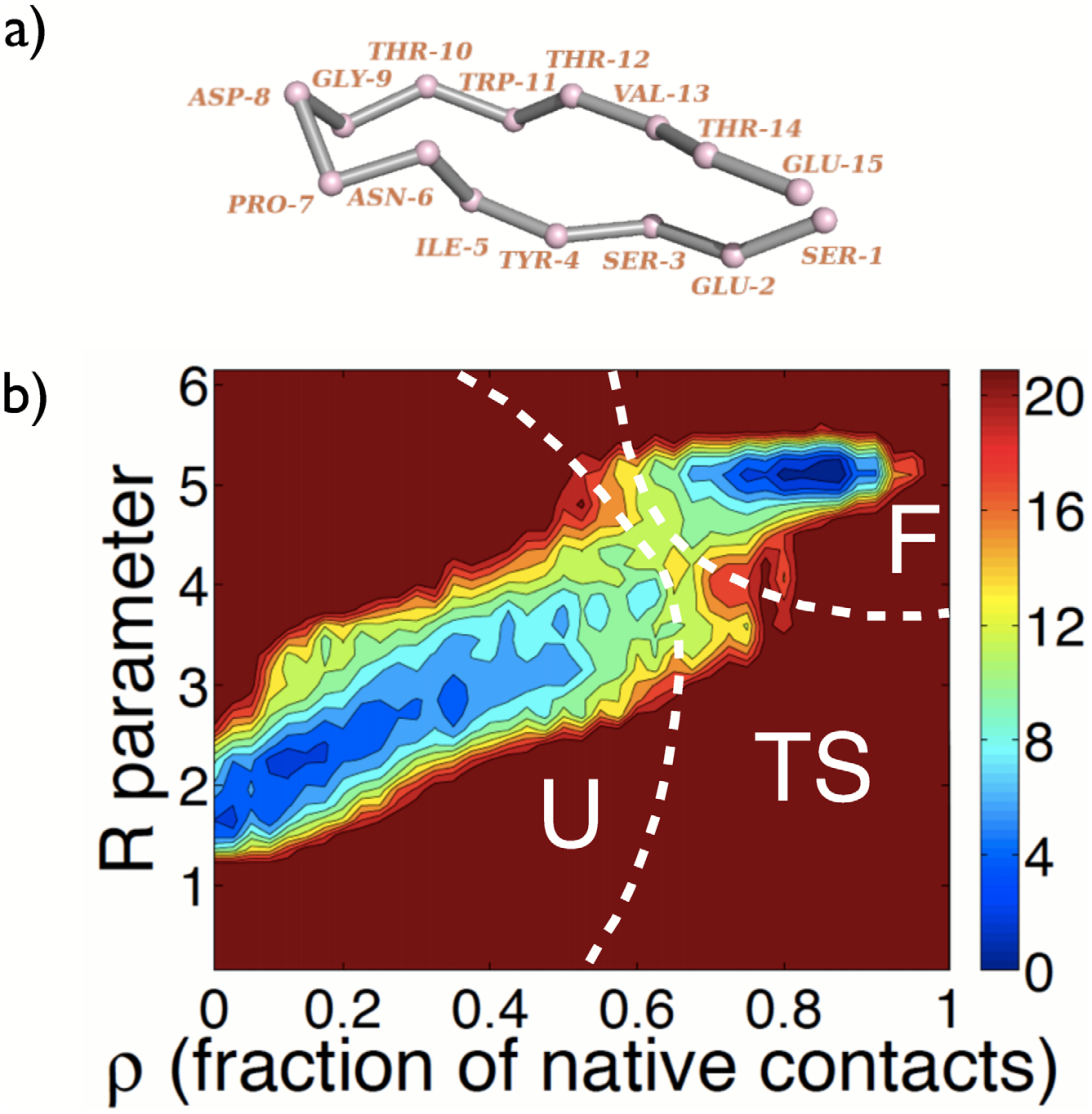
Snapshot of Peptide 1 and contour map of the free energy change. a) Backbone snapshot of Peptide 1 extracted at the end of the simulation started from the NMR structure. For clarity only the C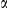 atoms are shown. b) Contour map of the free energy change, 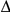
A, as a function of the position in the 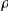-R plane (fraction of native contacts, 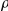, within the peptide - R parameter). 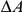 is calculated with respect to the state with the highest probability, i.e., the 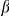-hairpin state. Three distinct states can be identified. The states with the two deep minima are the unfolded (U) and folded (F) states and the saddle point corresponds to the single transition state (TS), i.e., the barrier that all molecules must cross if they are to fold to the native state. The F, U and TS states were defined as follows: F comprises all structures populating the basin around the global minimum at 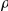 = 0.85 and R = 4.95, and with free energy values within 12 kJ/mol from the global minimum; U comprises all structures populating the basin around the local minimum at 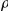 = 0.10 and R = 2.10, and also with free energy values up to 12 kJ/mol; the structures at the top of the barrier, i.e., not belonging to F or U, were assigned to the TS.

In the present study, we examine the transition state ensemble and folding dynamics of a model system, Peptide 1, a 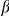-hairpin peptide of 15 residues (Figure 1a) [38]. Although Peptide 1 is a designed peptide, the turn sequence, NPDG, is statistically the most abundant type I turn in proteins, enhancing the relevance of the study of its folding mechanism for natural proteins [39]. This peptide has been found to fold via a two-state mechanism in 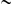
0.8 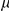
s, as determined by the T-jump technique in combination with IR [38], [40]. In our previous work, the folding kinetics of this peptide was examined using multiple independent 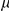
s-timescale all-atom MD trajectories in explicit solvent, yielding a folding time in accord with the above experimental datum [41].

Here, we derive the configurations of the TSE from the free energy folding landscape of Peptide 1 generated by multiple atomistic MD simulations over a total simulation time of 15 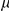 s. The trajectories were started from fully unfolded structures and several spontaneous folding events to the NMR-derived [38] 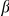-hairpin were observed, thus enabling a dynamical characterization of the evolution of the peptide through the folding TS. To our knowledge, this is the first time that multiple 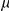
s-long explicit solvent, unbiased simulations with clear unfolding-folding transitions have been used to characterize structurally and dynamically the TS in folding studies.

The MD trajectories allow determination of the mechanism by which the TS is reached and subsequent events in folding pathways. The role of non-native interactions is characterized. It is found that, rather than being reached and crossed by highly-random fluctuations (i.e., through very different and heterogeneous pathways), folding is characterized by a directed, stagewise process involving the formation of specific structures before, during, and after the transition state for folding, corresponding to a structured folding pathway.

## Results

Six 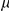
s-timescale atomistic MD simulations of Peptide 1 in explicit solvent (total of 15 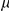 s) were performed, starting from unfolded structures, and folding to the native NMR-derived conformation [38] was observed in all of them [41]. The six trajectories were used to evaluate the free energy landscape of the system using two progress variables based on the native topology of the peptide: the R parameter, containing information on the backbone, and the fraction of native contacts (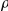) containing information on the sidechain packing (Figure 1b). These two order parameters were chosen in order to capture most of the information relevant to folding and are based on the native topology. Indeed, it has been shown that for two-state peptides, such as the present, global coordinates based on the native topology fully satisfy the criteria needed to accurately identify and describe TSEs [25]. The corresponding free energy map can be divided into three distinct regions: the folded state F, the unfolded state U and a single barrier, the transition state TS (see caption of the figure for the state definitions). Concerning the sensitivity of TS structures derived from the selected reaction coordinates, an additional free-energy landscape was calculated as a function of the RMSD-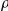 pair of variables. TS structures derived from the RMSD-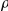 landscape were compared with the original TS structures of the R-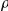 plane. The overlap between the two TSE is approximately 70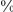, which is acceptable and further supports the choice of the original progress variables. The TSE was slightly refined. We have noted that in one of the folding transitions (along trajectory 6) that was initially used to define the TSE, the peptide actually folds to a conformation that falls into the F state definition (R = 4.8, 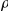 = 0.65), but is actually non-native, having a clearly non-native turn. This conformation does not populate the free energy minimum, but rather a region with a 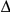
A value of 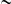
10 kJ/mol (see Figure S1). We, thus, excluded the TS structures sampled along this transition from the final TSE.

**Figure 2:**
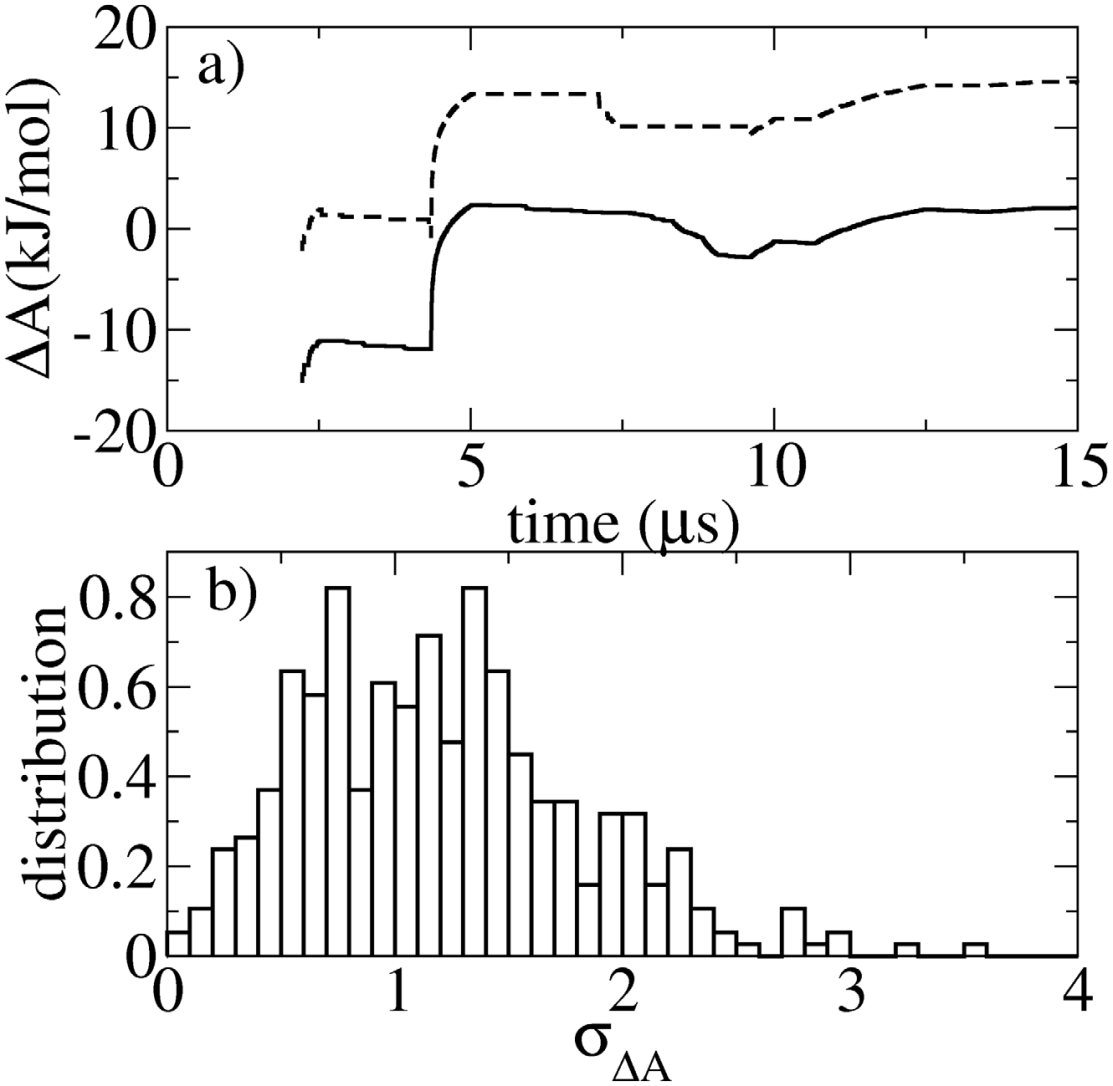
Convergence with time of the free energy change. a) Time convergence of the 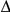
A for two typical grid cells in the 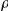-R free energy landscape: One cell in the U state around the local minimum with 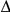
A 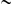 2 kJ/mol (solid line), and the other cell in the TS region with 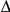
A 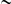 14 kJ/mol (dashed line). b) Probability distribution of the free energy standard deviations, 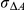, for all the grid cells of the free energy landscape.

To estimate the reliability of the free energy surface the convergence of the free energies associated with individual grid cells of the plane was examined (note that free energy values are defined with respect to the grid cell corresponding to the global minimum). Figure 2a shows a typical convergence plot for two given grid cells, one in the TS (Figure 2a shows a typical convergence plot for two given grid cells, one in the TS (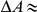 14 kJ/mol) and the other in the local minimum of the unfolded state (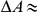 2 kJ/mol). After about 5–10 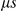 of sampling for all grid cells, the values are rather stable (within 1–2 kJ/mol). Figure 2b shows the probability distribution of the free energy standard deviations, 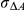 (see Methods section for the estimate of the errors), for all the grid cells. Again, these show relatively small statistical errors in the free energy values (again within 2 kJ/mol).

**Figure 3:**
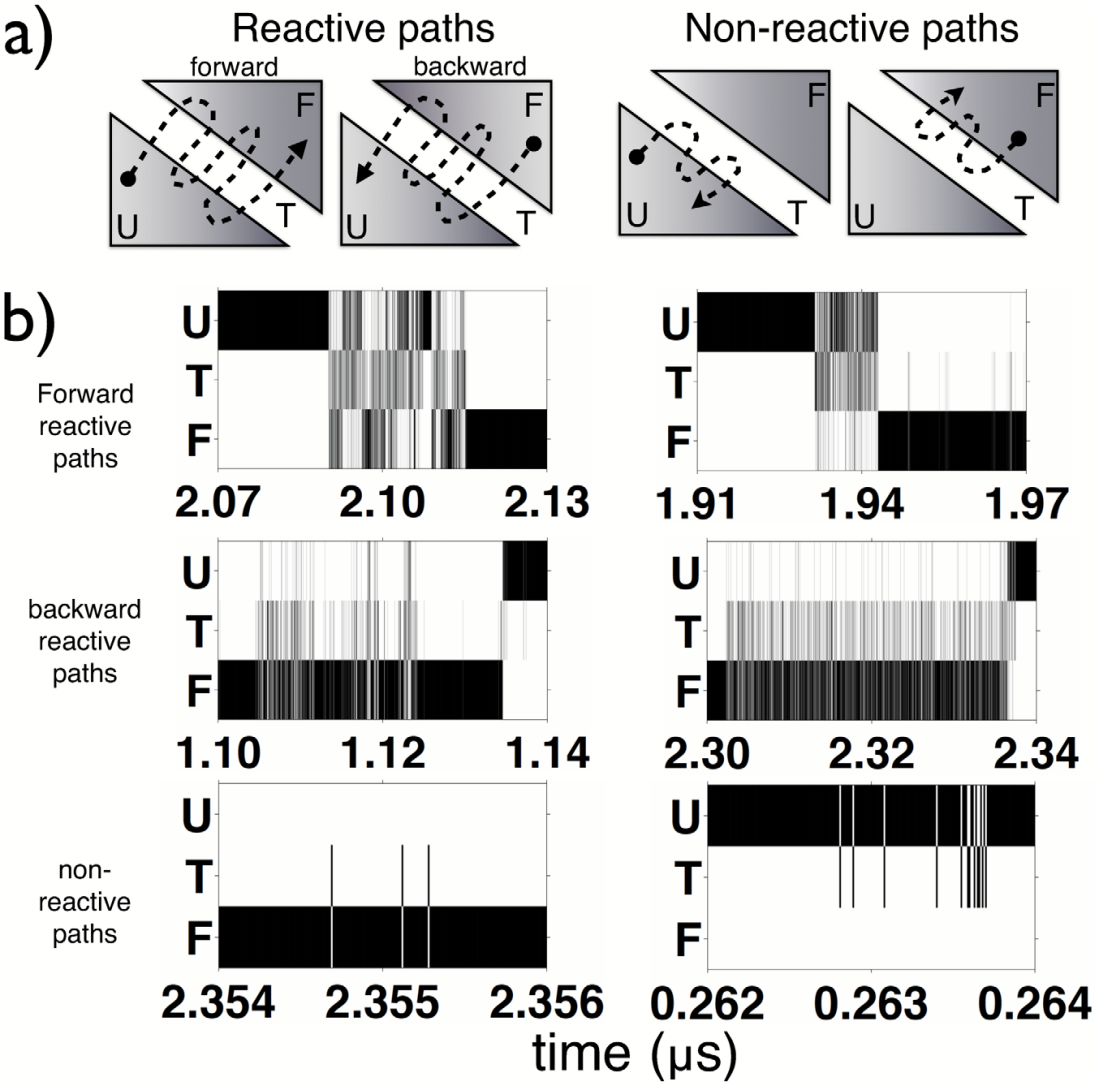
Scenarios for the path reaching and crossing the TS. a) Schematic diagram showing the possible scenarios for the paths reaching the TS, namely: reactive and non-reactive paths; in the reactive paths, the crossing of the TS can occur either from U to F (forward reactive path) or from F to U (backward reactive path); in non-reactive paths, the peptide falls back into the state of origin (either U
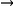
TS
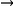
U or F
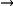
TS
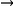
F paths). b) Time evolution of the peptide through the U, TS and F states for six representative paths along the simulations. The three rows in each panel represent the existence of the U (unfolded), T (transition) and F (folded) states. All the four scenarios described in panel a can be observed. The top and middle panels show the forward and backward reactive paths, respectively. The bottom panels show the non-reactive paths, either U
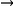
TS
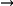
U or F
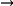
TS
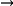
F paths.

Overall, the free energy landscape of Peptide 1 represents a typical two-state folder, consistent with the mono-exponential folding kinetics observed in both laser-induced temperature-jump experiments [40] and in our 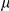
s MD simulations as reported previously [41]. Thus, the following minimal mechanism can be assumed:
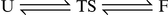



Four possible dynamical scenarios emerge (see Figure 3a): the peptide climbs from the unfolded basin to the TS and either descends forward to the folded state (forward reactive path U
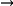
TS
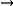
F) or falls back to the unfolded basin (non-reactive path U
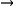
TS
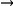
U); or the peptide climbs from the folded basin to the TS and either descends to the unfolded state (backward reactive path F
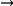
TS
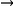
U) or falls back into the folded basin (non-reactive path F
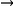
TS
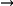
F).

The occurrence of conformations belonging to the TS, F and U states can be followed in time along the six unbiased MD trajectories. Examples of the dynamical evolution of the peptide through the three states are shown in Figure 3b. All four cases described above occur. In all four scenarios, once the TS is reached, fast recrossings of the TS surface are observed before the final descent to the end-state. Particularly remarkable is the fact that many of the TS structures, which were extracted based on a thermodynamic criterion, indeed occur right at the folding and unfolding transitions along the (unbiased) trajectories (top and middle panels of Figure 3b), thus confirming the validity of the thermodynamic TS selection. In addition, TS structures occurring in non-reactive paths (bottom panels of Figure 3b) are also observed, corresponding to very short excursions into the TS compared to reactive paths.

In what follows, the kinetics and structural features of the TSE are presented. Furthermore, to determine what triggers the folding events, the TS structures visited along forward reactive paths are examined in detail.

**Figure 4:**
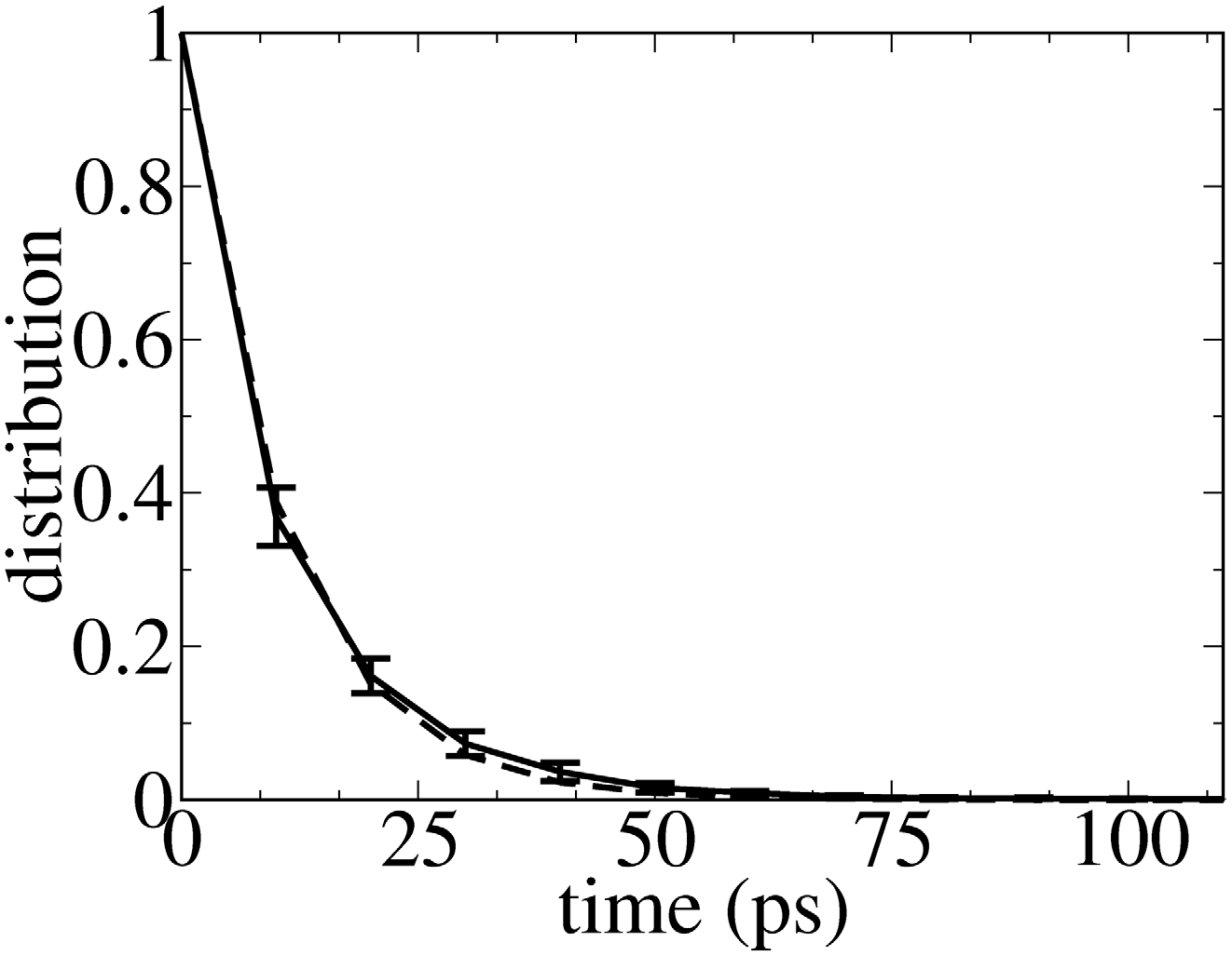
Distribution of TS lifetime. Distribution of TS lifetimes, defined as the mean residence time in TS, for Peptide 1 (solid line). The dashed line shows a mono-exponential fit to the data. The error bars represent one standard deviation. The time constant of the exponential fit is taken as the average lifetime of the TS.

To characterize the TS kinetics, the TS lifetime (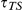), here defined as the mean residence time in the TS, was evaluated. The distribution of the residence times (Figure 4) was fitted by a monoexponential function (dotted line) yielding a 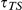 of 10.2
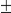
0.5 ps (for the estimate of the error see Methods), with 52.7% and 47.3% of the TS population ending up in the folded and unfolded states, respectively. The correlation coefficient is higher than 0.9999, showing the goodness of the fit. These data are consistent with a two-state kinetic model [42], thus further indicating that the selected TSE is reliable. The relatively short (ps-timescale) TS lifetime obtained, showing that the TS is a short-lived state, is consistent with the fast recrossings of the TS observed prior to the final descent (see Figure 3b).

**Figure 5:**
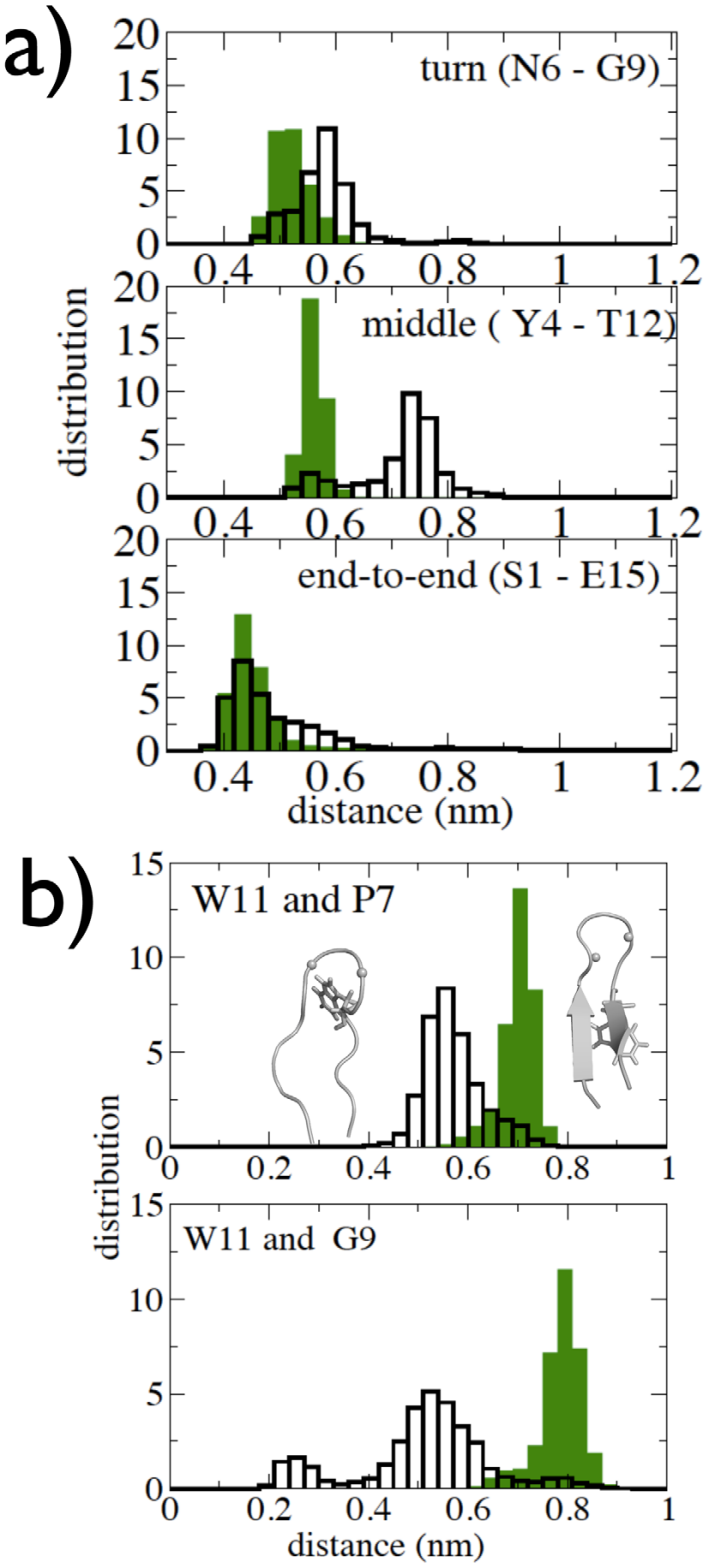
Distribution of turn, middle and end-to-end segments of Peptide 1. a) Distributions of the distances between the C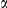 atoms of residues N6-G9 (top), I4-T12 (middle) and S1-E15(bottom) in the TSE. The corresponding distributions in the F state are shown as green filled bars. b) Distributions of the minimum distances between the atoms of the W11 sidechain and the atoms of the sidechain of two turn residues, namely P7 (top) and G9 (bottom) in the TSE. The corresponding distributions in the F state are shown as green filled bars. Snapshot of TS structure at left shows the key-lock configuration of W11 with P7 and G9 residues, while folded structure at the right has W11 in native configuration.

In order to examine the degree of native topology in the TSE, the formation of turn, middle and end-to-end inter-strand contacts was evaluated and compared with the corresponding contacts in the folded conformations. Figure 5a shows the distributions of the distances between the C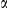 atoms of residues S1-E15 (end), I4-T12 (middle) and N6-G9 (turn) in the TSE. The distributions are quite narrow, indicative of a rather homogeneous topology of the TSE population. However, a minor peak is present in the middle distribution at 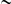
0.55 nm that will be discussed below. The “end” and “turn” distributions in the TSE, peaked at 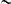
0.42 and 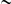
0.55, respectively, are native-like, the only slight difference being a shift of the “turn” peak maximum to longer values (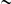
0.55 nm versus 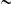
0.60 nm in F and TSE, respectively). In contrast, the middle-contacts distribution differs significantly in the two states, the peak maximum being at a much longer distance in the TSE (0.72 nm) than in F (0.54 nm). Thus, the distinct feature of most of the TSE is the formation of native-like end-to-end and turn contacts, while the central parts of the strands are disordered. The presence of the end-to-end contact is consistent with recent experimental results on several proteins suggesting that the TS exhibits an overall native-like topology in which the N-terminal and C-terminal regions are in close proximity [17], [32], [43]. As was found for the backbone topology, the native sidechain contacts at the end of the hairpin are present in the TSE (data not shown). Contrarily, the sidechains of the central parts exhibit some persistent non-native interactions. In particular, W11, the bulkiest of the sidechains, forms non-native contacts with two residues of the turn, namely P7 and G9, in a kind of key-lock configuration (see Figure 5b, in which the distributions are plotted of the minimum distances between the W11 sidechain and the P7 and G9 sidechains, together with representative configurations).

To analyze the origin of the above-mentioned minor peak in the “middle” distribution, possible differences in the topological features of TS structures sampled along reactive (U
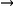
TS
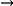
F and F
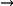
TS
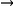
U) and non-reactive (F
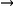
TS
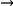
F and U
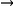
TS
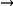
U) pathways were investigated. Separating the TSE into U
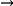
TS
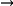
F, F
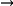
TS
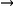
U, F
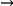
TS
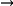
F and U
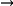
TS
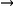
U structures is also useful for identifying the presence, if any, of a specific folding nucleus, i.e., a nucleus of contacts resulting in rapid assembly of the native state [24], [44], thus triggering the descent of the peptide from the TS to the folded basin. The folding nucleus is more likely to be retained in the F
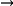
TS
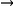
F conformations than in the U
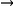
TS
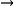
U conformations [24], [44].

**Figure 6:**
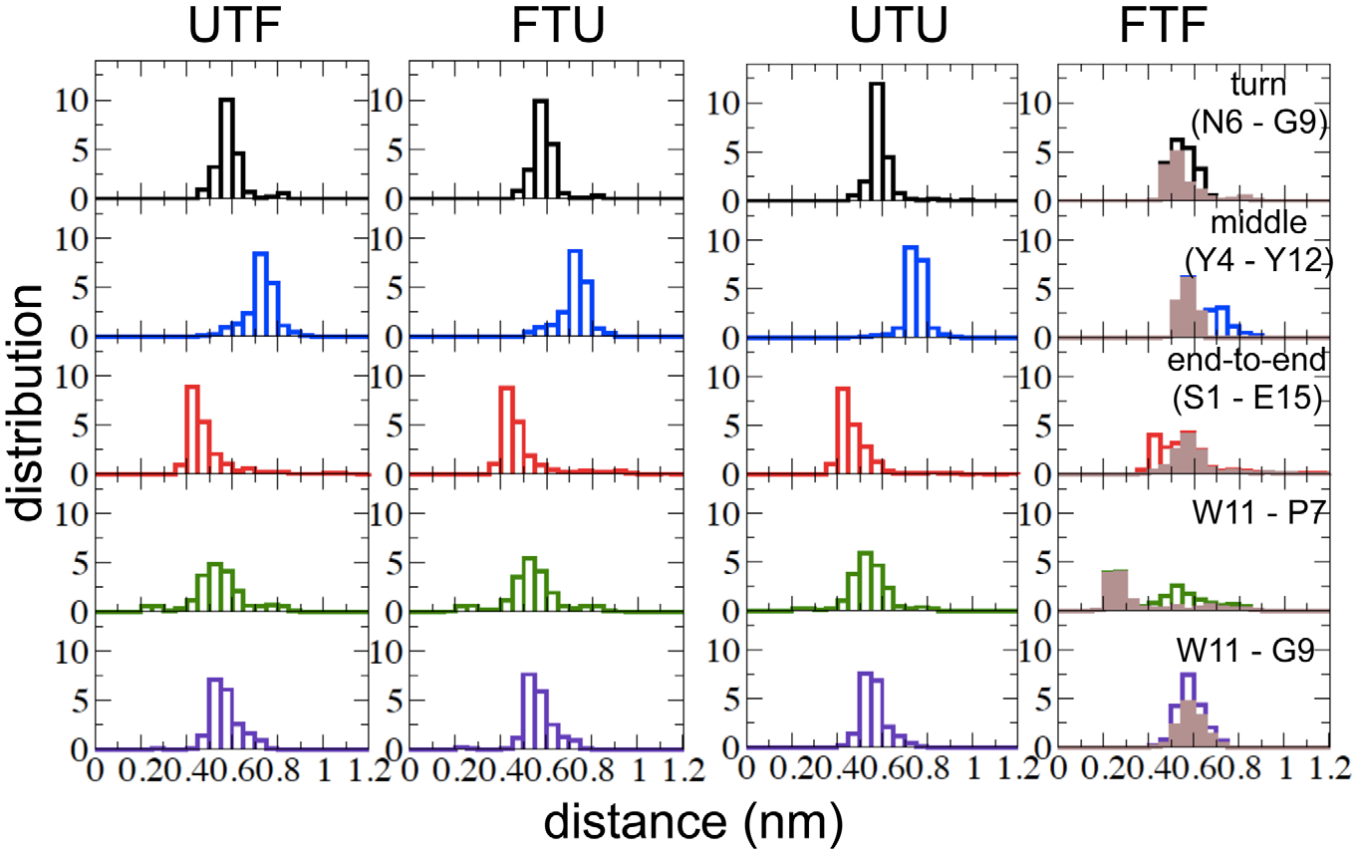
Distribution of various topological features in U-TS-F, F-TS-U, U-TS-U and F-TS-F subpopulations of the TSE of Peptide 1. Distributions of the distance between the C-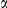 atoms of the residues N6-G9 (first row), Y4-Y12 (second row), S1-E15 (third row). Distributions of the minimum distances between the atoms of the W11 sidechain and the atoms of the sidechain of two turn residues, namely P7 (fourth row) and G9 (fifth row). The F-TS-F subpopulation shown in brown color corresponds to the structures with middle backbone contacts formed.

Therefore, we calculated the distribution of “turn”, “middle”, “end-to-end” and W11-P7/W11-G9 contacts in the TS structures belonging to U
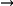
TS
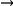
F, F
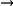
TS
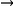
U, F
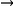
TS
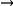
F and U
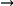
TS
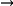
U paths separately (see Figure 6). It is found that, TS structures apart from the F
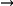
TS
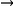
F population are very homogeneous, with the topological features described above (i.e., the turn and end-to-end contacts are formed, the central part of the backbone is disordered and the W11 sidechain is positioned in the middle of the turn at around 0.55 nm from P7 and G9). In contrast, however, in about 65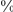 of the F
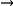
TS
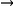
F structures (brown color in Figure 6), which correspond to the minor peak mentioned above, the middle backbone contacts are formed, the end-to-end contacts is slightly looser and the W11 sidechain is closer to P7 (at a distance of 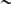
0.3 nm) than to G9. Hence, according to the definition given above, the folding nucleus is likely to be characterized by an almost-native backbone topology and a persistent, non-native sidechain contact between W11 and the P7 turn residue.

**Figure 7:**
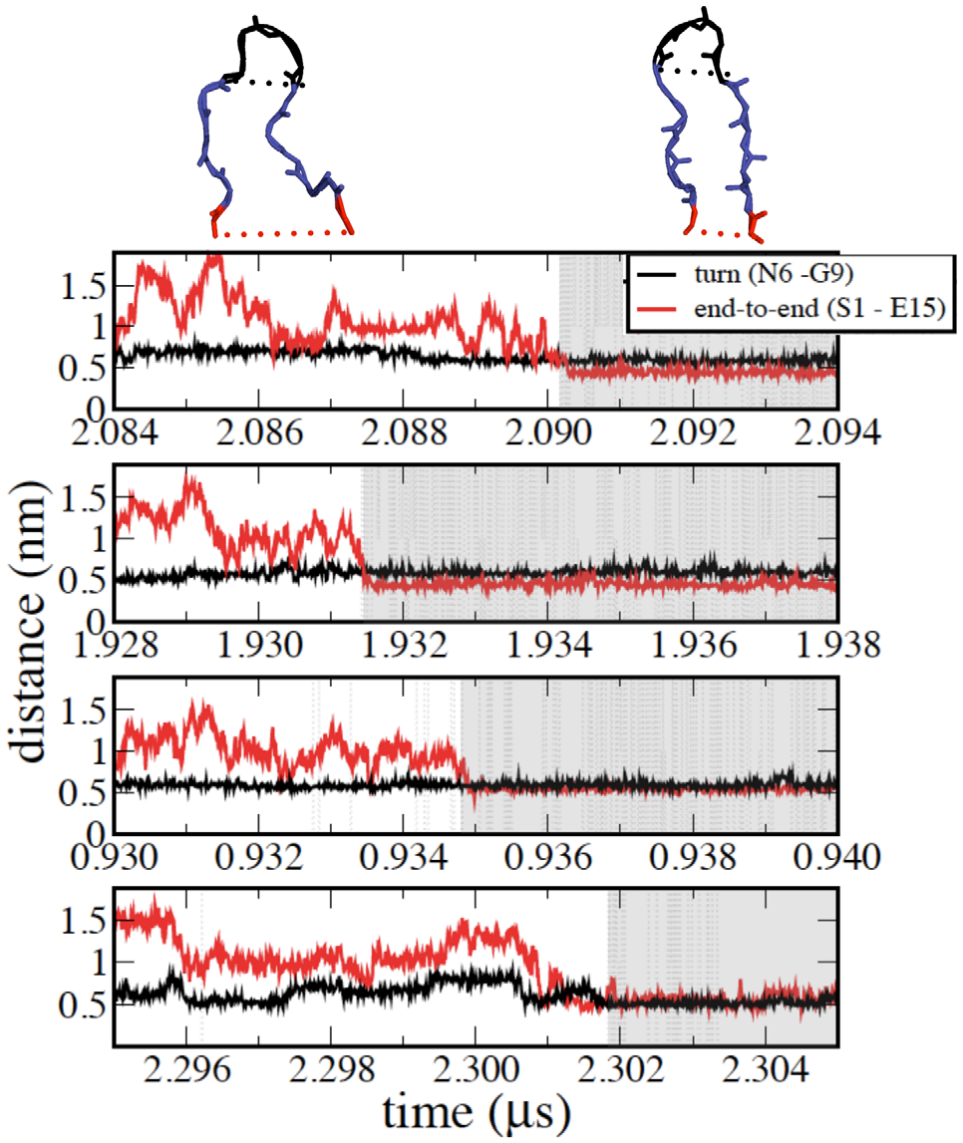
Representative forward reactive paths illustrating the events occurring prior to reaching the TS. The TS region is shaded in grey. The time evolution of the distance between the C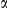 atoms of the turn (N6-G9) and end (S1-E15) residues are plotted in black and red, respectively.

The mechanism by which the peptide crosses the TS is now examined. To determine whether the end and turn contacts are formed only at the TS or prior to it, the time evolution of the corresponding C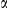-C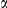 distances was calculated. Figure 7 shows these time series for four representative forward reactive paths. Clearly, prior to TS the turn is already formed, while the end-to-end contact forms only at the TS. These results indicate that end-to-end contact formation is a discriminating feature for the TSE of Peptide 1. This trend is observed in all forward reactive paths.

**Figure 8:**
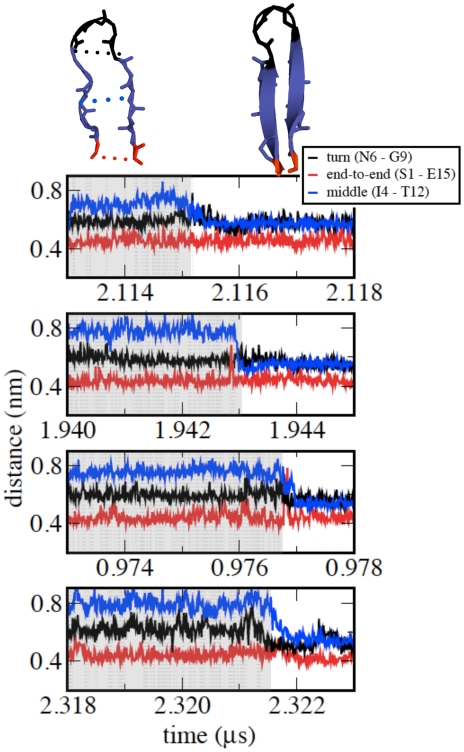
Representative forward reactive paths illustrating the events occurring after the TS. The TS region is shaded in grey. The time evolution of the distance between the C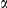 atoms of the turn (N6-G9), end (S1-E15) and middle (I4-T12) residues are plotted in black, red and blue, respectively.

The final stage of the folding process, i.e, the descent of the peptide from the TS to the F state, involves the correct arrangement of the middle part of the hairpin. Figure 8 plots the C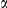-C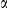 distances of the end (S1-E15), middle (Y4-T12) and turn (N6-G9) residues again as a function of time for four representative reactive folding paths. The end and turn regions of the hairpin remain in contact during the final stage, while the middle part comes into contact.

**Figure 9:**
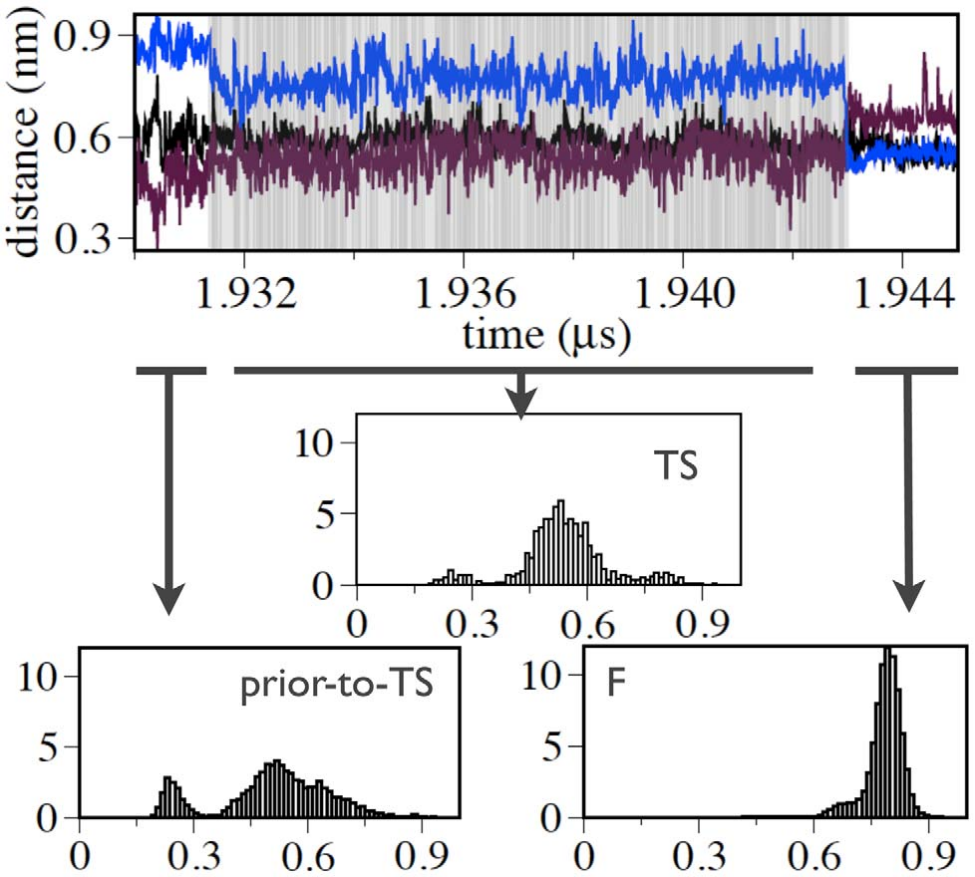
Time evolution of the minimum distance between all the atoms of the W11 sidechain and all the atoms of the P7 sidechain (maroon) for a representative reactive path. In addition, the distances between the C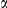 atoms of the turn (N6-G9) and central (I4-T12) residues are shown in black and blue, respectively. The shaded region represents the TS. The three histograms at the bottom show the distributions of the minimum distances between the W11 and P7 sidechains for the structures sampled before the TS (prior-to-TS), in the TS (TS) and in the native (F) state evaluated over all forward reactive paths. For the prior-to-TS ensemble the structures occurring within 1 ns prior to the TS along all forward reactive paths are taken.

Finally, the time evolution of the non-native contacts formed by the W11 sidechain with the turn is analyzed. These contacts are present already prior to the reaching the TS, locking the P7-G9 residues into the “reactive” turn conformation (see Figure 9). In the final stage of the process, the W11 sidechain loses the non-native contacts with the turn, allowing a concomitant rearrangement of the central segments of the strands into the native 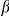-hairpin conformation (see Figure 9).

**Figure 10:**
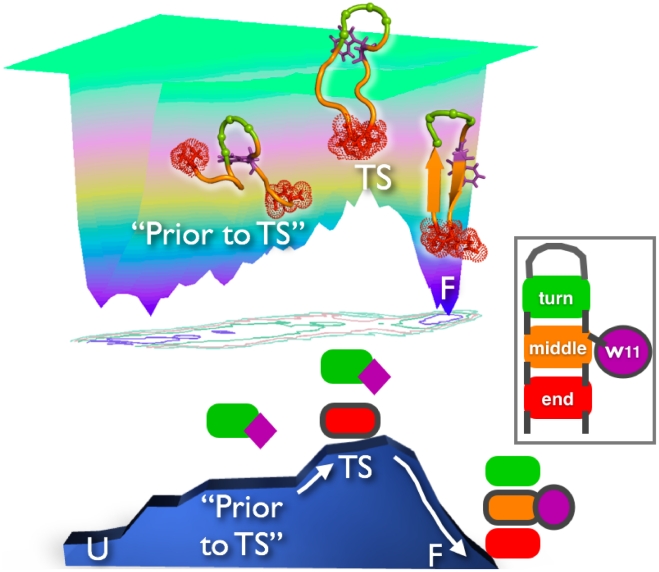
Three-dimensional representation of folding funnel of Peptide 1. The mechanism by which the peptide crosses TS is shown. The TS is reached with the formation of native end-to-end (red) and turn (green) contacts, with the end-to-end formation appearing at the TS, making it the unique feature of the TS of Peptide 1. However, reaching the folded state from the TS involves W11 sidechain (purple) repositioning and concomitant native arrangement of the central segment (orange) of 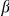-hairpin. In “Prior-to-TS” and TS, W11 is in a ‘key-lock’ configuration with turn residues, thus from a dynamic point of view it acts as a chaperone to reach the TS, while the last stage to reach the native state involves ‘correct’ native arrangement of W11 with the central segment of 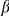-hairpin (See representative structures).

The above results indicate a clear TS folding mechanism that is summarized in Figure 10. In this mechanism, the TS is reached with the formation of end-to-end and turn contacts, with the turn appearing prior to the TS and the end-to-end interaction appearing only at the TS. Reaching the folded state from the TS requires W11 sidechain repositioning and concomitant native rearrangement of the central segment of 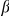-hairpin. However, despite a common TS topology and a single, directed folding-transition mechanism was observed from extensive MD simulation, the existence of minor folding pathways cannot be excluded.

## Discussion

A variety of computational models and methods has been applied to extract information on the TSE for protein folding, including Go models [6], [10], [45], high temperature [14] and implicit solvent [15], [16] MD simulations, and transition path sampling [4], [5]. However, the uncertainty involved in these methods (e.g., unnaturally high temperature or absence of explicit water) leads to uncertainty in the interpretation of the data [46]. To our knowledge, no simulation studies exist that have characterized the TSE using unbiased atomistic simulation in explicit solvent, in which folding events from fully unfolded conformations occur. In the present work, we have used this information to directly evaluate TS structure and kinetics from multiple 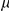
s-timescale all-atom explicit MD simulations of the 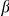-hairpin Peptide 1.

The main findings of the study can be summarized as follows. Structurally, the TS consists of a rather homogeneous loop-like topology characterized by native end-to-end contacts and specific non-native interactions in the middle region of the loop characterized by the W11 sidechain centrally locked in between the turn residues P7 and G9. A specific folding nucleus, i.e., the nucleus of contacts resulting in rapid assembly of the native state, was also identified. This is characterized by loose end-to-end contacts and the presence of native contacts in the middle part of the hairpin, with a concomitant shift of the W11 sidechain towards the sidechain of P7.

Concerning the folding mechanism, while the turn and its non-native contacts with the W11 sidechain are already formed prior to the TS, the end-to-end interaction appears only at the TS, making it a unique feature of the TS. The TS is short-lived, with a mean lifetime on the ps-timescale. The final stage in reaching the native state, occurring after crossing the TS, involves the cooperative loss of the non-native contacts and formation of the native inter-strand contacts in the central part of the hairpin. These last events are those committing the reactive trajectories to very rapidly proceeding from TS to F.

The present results support previous experimental findings on other systems suggesting that the formation of end-to-end contacts in the TS may be a fairly general phenomenon in the folding of small proteins [17], [32], [43]. In the present case, there exists an additional structural feature specific to 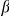-hairpins that further restricts the conformational variety of the TS structures, namely the formation of the turn.

From a dynamical point of view, W11 acts as a chaperone for reaching the TS through the formation of non-native interactions with the turn. The W sidechain locks the turn in place prior to TS, allowing the ends to subsequently come into contact, i.e., the TS to be reached. Hence, also from a dynamical point of view our results are in favour of a “directed”, stagewise process [11], rather than a large number of heterogeneous pathways [47], [48] characterizing the reaching and crossing of the TS. Further studies on other peptides and proteins will clarify the generality of the present observation of the structuring of the pathway across the transition state for folding.

## Methods

### Molecular dynamics simulations

A series of six 2.5 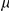
s-long atomistic MD simulations of Peptide 1 (SESYINPDGTWTVTE) in explicit solvent was performed [41]. Six starting structures representing the unfolded state were extracted randomly from a simulation of 50 ns that was started from a fully extended conformation of the peptide. The MD simulations were performed with the program GROMACS [49] with the OPLS-AA all-atom force field [50] for the peptide. The water was modelled using the TIP4P representation [51]. Each of the six starting conformations was placed in a dodecahedral water box large enough to contain the peptide and at least 1.0 nm of solvent on all sides (volume
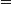
48 nm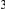). Each simulation box contained 6647 atoms. Periodic boundary conditions were applied and the long range electrostatic interactions were treated with the Particle Mesh Ewald method [52] using a grid spacing of 0.12 nm combined with a fourth-order B-spline interpolation to compute the potential and forces in-between grid points. The real space cut-off distance was set to 0.9 nm and the van der Waals cut-off to 1.4 nm. The bond lengths were fixed [53] and a time step of 2 fs for numerical integration of the equations of motion was used. Simulations were performed in the NVT ensemble with isokinetic temperature coupling [54] keeping the temperature constant at 300 K. Three Na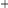 counterions were added, replacing three water molecules, so as to produce a neutral simulation box.

All the starting structures were subjected to a two-stage energy minimization protocol using the steepest descent method. The first minimization was performed with the coordinates of the peptide held fixed, allowing only the water and the ions to move, and the second was performed on the atoms of both the peptide and the solvent molecules. The temperature of the system was then increased from 50 K to 300 K in 500 ps of MD before the 2.5 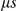 production simulations were started. For all the analyses conformations saved every 10 ps were used.

### Analysis of trajectories

The trajectories were analyzed using order parameters that capture principal aspects of the folding process of the peptide. A robust parameter for identifying conformational transitions is ‘R’ [55], calculated as follows:
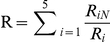
where R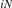 is the i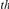 inter-strand C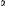-C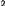 distance in the native NMR structure and R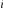 is the same distance in the MD. The five inter-strand C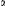-C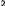 pairs in the Peptide 1 hairpin are the following: N6-T10, I5-W11, Y4-T12, S3-V13 and E2-T14. A value of R
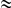
5 indicates formation of the native 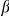-hairpin. Sidechain packing was quantified via the fraction of native sidechain contacts, 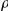 (a contact between two sidechains is considered to be formed when the minimum distance between the atoms belonging to the sidechains is 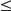
0.55 nm).

Given a system in thermodynamic equilibrium, the change in free energy on going from a reference state, *ref*, of the system to a generic state, *i*, (e.g., from unfolded to folded) at constant temperature and constant volume was evaluated as
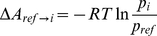
(1)where *R* is the ideal gas constant, *T* is the temperature and *p* 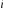 and *p* 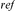 are the probabilities of finding the system in state *i* and state *ref*, respectively. We describe the free energy surface as a function of two order parameters, namely the fraction of native contacts, 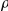, and the R parameter. A grid 40×40 was used to divide this plane in 1600 cells and for every cell the number of points counted and the relative probability calculated, allowing 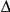
A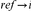 to be determined. The reference state was chosen to be the grid cell with the highest probability, which corresponds to folded 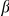-hairpin structures.

The statistical error in different properties evaluated from the simulations, such as the TS lifetime or the free energy values, was estimated through the standard error of their mean, 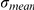, calculated over 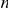 subsets of the trajectories:
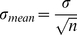
(2)

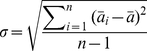
(3)

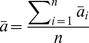
(4)where 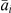 is the mean of the given parameter evaluated in the i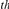 subset, and 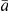 and 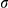 are the mean value over the 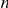 samples and the sample standard deviation, respectively. Here we used 3 independent subsets of the trajectories (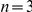, *i.e.* 3 groups each consisting of two trajectories), which was found to be a good compromise between the statistics within each subset and the sample size, 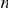. Assuming, as usual, a normal distribution of the mean value, 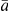, the expected value of 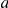 lies with 95% confidence inside the 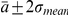 interval.

## Supporting Information

Figure S1
**TSE refinement.** a) Time evolution of R parameter (above), 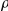 (middle) and distance between the C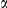 atoms of the turn N6-G9 (below) during one of the folding transitions along trajectory 6. The transition to the putative folded state is marked with the bold black line. b) The location on the free energy landscape of the putative folded structures shown in panel a) is highlighted with a black box.(PDF)

## References

[1] Abkevich VI, Gutin AM, Shakhnovich EI (1994). Specific nucleus as the transition state for protein folding: evidence from the lattice model.. Biochemistry.

[2] Shakhnovich E (2009). Protein folding roller coaster, one molecule at a time.. Proc Natl Acad Sci U S A.

[3] Daggett V, Fersht A (2003). The present view of the mechanism of protein folding.. Nat Rev Mol Cell Biol.

[4] Bolhuis PG, Chandler D, Dellago C, Geissler PL (2002). Transition path sampling: throwing ropes over rough mountain passes, in the dark.. Annu Rev Phys Chem.

[5] Bolhuis PG (2003). Transition-path sampling of beta-hairpin folding.. Proc Natl Acad Sci U S A.

[6] Ding F, Guo W, Dokholyan NV, Shakhnovich EI, Shea JE (2005). Reconstruction of the src-sh3 protein domain transition state ensemble using multiscale molecular dynamics simulations.. J Mol Biol.

[7] Pande VS, Rokhsar DS (1999). Folding pathway of a lattice model for proteins.. Proc Natl Acad Sci U S A.

[8] Winter G, Fersht AR, Wilkinson AJ, Zoller M, Smith M (1982). Redesigning enzyme structure by site-directed mutagenesis: tyrosyl trna synthetase and atp binding.. Nature.

[9] Fersht AR, Leatherbarrow RJ, Wells TN (1987). Structure-activity relationships in engineered proteins:analysis of use of binding energy by linear free energy relationships.. Biochemistry.

[10] Hubner IA, Edmonds KA, Shakhnovich EI (2005). Nucleation and the transition state of the sh3 domain.. J Mol Biol.

[11] Shimada J, Shakhnovich EI (2002). The ensemble folding kinetics of protein g from an all-atom monte carlo simulation.. Proc Natl Acad Sci U S A.

[12] Vendruscolo M, Paci E, Dobson CM, Karplus M (2001). Three key residues form a critical contact network in a protein folding transition state.. Nature.

[13] Gsponer J, Caflisch A (2002). Molecular dynamics simulations of protein folding from the transition state.. Proc Natl Acad Sci U S A.

[14] Chong LT, Snow CD, Rhee YM, Pande VS (2005). Dimerization of the p53 oligomerization domain: identification of a folding nucleus by molecular dynamics simulations.. J Mol Biol.

[15] Settanni G, Rao F, Caflisch A (2005). Phi-value analysis by molecular dynamics simulations of reversible folding.. Proc Natl Acad Sci U S A.

[16] Salvatella X, Dobson CM, Fersht AR, Vendruscolo M (2005). Determination of the folding transition states of barnase by using phii-value-restrained simulations validated by double mutant phiij-values.. Proc Natl Acad Sci U S A.

[17] V'arnai P, Dobson CM, Vendruscolo M (2008). Determination of the transition state ensemble for the folding of ubiquitin from a combination of phi and psi analyses.. J Mol Biol.

[18] Periole X, Vendruscolo M, Mark AE (2007). Molecular dynamics simulations from putative transition states of alpha-spectrin sh3 domain.. Proteins.

[19] Lindorff-Larsen K, Vendruscolo M, Paci E, Dobson CM (2004). Transition states for protein folding have native topologies despite high structural variability.. Nat Struct Mol Biol.

[20] Lam AR, Borreguero JM, Ding F, Dokholyan NV, Buldyrev SV (2007). Parallel folding pathways in the sh3 domain protein.. J Mol Biol.

[21] Hubner IA, Shimada J, Shakhnovich EI (2004). Commitment and nucleation in the protein g transition state.. J Mol Biol.

[22] Shea JE, Onuchic JN, Brooks CL (2002). Probing the folding free energy landscape of the src-sh3 protein domain.. Proc Natl Acad Sci U S A.

[23] Weikl TR, Palassini M, Dill KA (2004). Cooperativity in two-state protein folding kinetics.. Protein Sci.

[24] Ding F, Dokholyan NV, Buldyrev SV, Stanley HE, Shakhnovich EI (2002). Direct molecular dynamics observation of protein folding transition state ensemble.. Biophys J.

[25] Cho SS, Levy Y, Wolynes PG (2006). P versus q: structural reaction coordinates capture protein folding on smooth landscapes.. Proc Natl Acad Sci U S A.

[26] Rao F, Caflisch A (2004). The protein folding network.. J Mol Biol.

[27] Ozkan SB, Dill KA, Bahar I (2003). Computing the transition state populations in simple proteinmodels.. Biopolymers.

[28] Du R, Pande VS, Grosberg AY, Tanaka T, Shakhnovich ES (1998). On the transition coordinate for protein folding.. J Chem Phys.

[29] Fersht AR (1993). The sixth datta lecture. protein folding and stability: the pathway of folding of barnase.. FEBS Lett.

[30] Daggett V, Li A, Itzhaki LS, Otzen DE, Fersht AR (1996). Structure of the transition state for folding of a protein derived from experiment and simulation.. J Mol Biol.

[31] Fersht AR (2000). Transition-state structure as a unifying basis in protein-folding mechanisms: contact order, chain topology, stability, and the extended nucleus mechanism.. Proc Natl Acad Sci U S A.

[32] Itzhaki LS, Otzen DE, Fersht AR (1995). The structure of the transition state for folding of chymotrypsin inhibitor 2 analysed by protein engineering methods: evidence for a nucleationcondensation mechanism for protein folding.. J Mol Biol.

[33] Fowler SB, Clarke J (2001). Mapping the folding pathway of an immunoglobulin domain: structural detail from phi value analysis and movement of the transition state.. Structure.

[34] Moran LB, Schneider JP, Kentsis A, Reddy GA, Sosnick TR (1999). Transition state heterogeneity in gcn4 coiled coil folding studied by using multisite mutations and crosslinking.. Proc Natl Acad Sci U S A.

[35] Daggett V, Fersht AR (2003). Is there a unifying mechanism for protein folding?. Trends Biochem Sci.

[36] Dinner AR, Karplus M (1999). Is protein unfolding the reverse of protein folding? a lattice simulation analysis.. J Mol Biol.

[37] Finkelstein AV (1997). Can protein unfolding simulate protein folding?. Protein Eng.

[38] Santiveri CM, Rico M, Jim'enez MA (2001). 13c(alpha) and 13c(beta) chemical shifts as a tool to delineate beta-hairpin structures in peptides.. J Biomol NMR.

[39] Wilmot CM, Thornton JM (1988). Analysis and prediction of the different types of beta-turn in proteins.. J Mol Biol.

[40] Xu Y, Oyola R, Gai F (2003). Infrared study of the stability and folding kinetics of a 15-residue beta-hairpin.. J Am Chem Soc.

[41] Thukral L, Smith JC, Daidone I (2009). Common folding mechanism of a beta-hairpin peptide via non-native turn formation revealed by unbiased molecular dynamics simulations.. J Am Chem Soc.

[42] Amadei A, D'Abramo M, Ramondo F, Daidone I, D'Alessandro M (2006). Statistical mechanical characterization of the free energy surface and classical kinetics for an intramolecular reaction: proton transfer in aqueous malonaldehyde.. J Phys Org Chem.

[43] McCallister EL, Alm E, Baker D (2000). Critical role of beta-hairpin formation in protein g folding.. Nat Struct Biol.

[44] Dokholyan NV, Buldyrev SV, Stanley HE, Shakhnovich EI (2000). Identifying the protein folding nucleus using molecular dynamics.. J Mol Biol.

[45] Karanicolas J, Brooks CL (2003). Improved go-like models demonstrate the robustness of protein folding mechanisms towards non-native interactions.. J Mol Biol.

[46] Rhee YM, Sorin EJ, Jayachandran G, Lindahl E, Pande VS (2004). Simulations of the role of water in the protein-folding mechanism.. Proc Natl Acad Sci U S A.

[47] Klimov DK, Thirumalai D (1998). Lattice models for proteins reveal multiple folding nuclei for nucleation-collapse mechanism.. J Mol Biol.

[48] Klimov DK, Thirumalai D (2001). Multiple protein folding nuclei and the transition state ensemble in two-state proteins.. Proteins.

[49] Spoel DVD, Lindahl E, Hess B, Groenhof G, Mark AE (2005). Gromacs: fast, flexible, and free.. J Comput Chem.

[50] Jorgensen WL, Tirado-Rives J (1988). The opls force field for proteins: Energy minimizations for crystals of cyclic peptides and crambin.. J Am Chem Soc.

[51] Jorgensen WL, Chandrasekhar J, Madura JD (1983). Comparison of simple potential functions for simulating liquid water.. J Chem Phys.

[52] Darden T, York D, Pedersen L (1993). Particle mesh Ewald: An N-log(N) method for Ewald sums in large systems.. J Chem Phys.

[53] Hess B, Bekker H, Berendsen HJC, Fraaije JGEM (1997). Lincs: A linear constraint solver for molecular simulations.. J Comp Chem.

[54] Brown D, Clarke JHR (1984). A comparison of constant energy, constant temperature, and constant pressure ensembles in molecular dynamics simulations of atomic liquids.. Mol Phys.

[55] Yang S, Onuchic JN, García AE, Levine H (2007). Folding time predictions from all-atom replica exchange simulations.. J Mol Biol.

