# Supplementary material for: Structured Pathway across the Transition State for Peptide Folding Revealed by Molecular Dynamics Simulations
Source: PLoS Comput Biol. 2011 Sep 8;7(9):e1002137. doi: 10.1371/journal.pcbi.1002137 (PMC3169518; doi:10.1371/journal.pcbi.1002137)
Supplement: Figure S1 — TSE refinement. a) Time evolution of R parameter (above), (middle) and distance between the C atoms of the turn N6-G9 (below) during one of the folding transitions along trajectory 6. The transition to the putative folded state is marked with the bold black line. b) The location on the free energy landscape of the putative folded structures shown in panel a) is highlighted with a black box. (PDF) [file pcbi.1002137.s001.pdf]

Supplementary Information

Figure S1

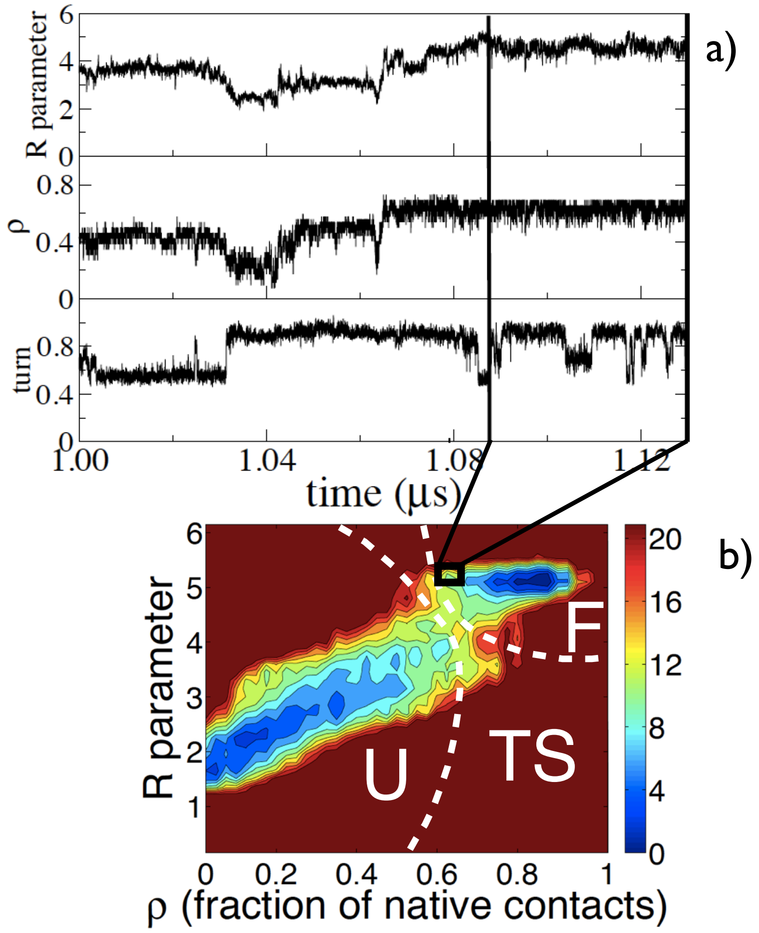

TSE Refinement. a) Time evolution of R parameter (above), ρ (middle) and distance between the Cα atoms of the turn N6-G9 (below) during one of the folding transitions along trajectory 6. The transition to the putative folded state is marked with the bold black line. b) The location on the free energy landscape of the putative folded structures shown in panel a) is highlighted with a black box.
